# Nonlinear Muscles, Passive Viscoelasticity and Body Taper Conspire To Create Neuromechanical Phase Lags in Anguilliform Swimmers

**DOI:** 10.1371/journal.pcbi.1000157

**Published:** 2008-08-29

**Authors:** T. McMillen, T. Williams, P. Holmes

**Affiliations:** 1 Department of Mathematics, California State University Fullerton, Fullerton, California, United States of America; 2 Basic Medical Sciences, St. George's University of London, London, United Kingdom; 3 Program in Applied and Computational Mathematics, Princeton University, Princeton, New Jersey, United States of America; 4 Department of Mechanical and Aerospace Engineering, Princeton University, Princeton, New Jersey, United States of America; University of Washington, United States of America

## Abstract

Locomotion provides superb examples of cooperation among neuromuscular systems, environmental reaction forces, and sensory feedback. As part of a program to understand the neuromechanics of locomotion, here we construct a model of anguilliform (eel-like) swimming in slender fishes. Building on a continuum mechanical representation of the body as an viscoelastic rod, actuated by a traveling wave of preferred curvature and subject to hydrodynamic reaction forces, we incorporate a new version of a calcium release and muscle force model, fitted to data from the lamprey *Ichthyomyzon unicuspis*, that interactively generates the curvature wave. We use the model to investigate the source of the difference in speeds observed between electromyographic waves of muscle activation and mechanical waves of body curvature, concluding that it is due to a combination of passive viscoelastic and geometric properties of the body and active muscle properties. Moreover, we find that nonlinear force dependence on muscle length and shortening velocity may reduce the work done by the swimming muscles in steady swimming.

## Author Summary

In this article we develop a computationally tractable model for swimming in animals such as eels, lampreys, and aquatic snakes. The model combines motoneuronal activation, muscle dynamics, passive elasticity and damping in the spinal cord and body tissues, and simplified hydrodynamic reaction forces, thus allowing us to probe how neuromechanical interactions give rise to body shapes and, ultimately, motion through the water. We use it to investigate the sources of an interesting experimental observation in freely swimming fish: that waves of curvature propagating along the body lag behind and travel more slowly than the muscular activation waves measured by electromyography. By selectively “lesioning” components of the model, we deduce that the speed difference, at least in this type of fish, is largely due to passive viscoelasticity and body geometry. We also find that nonlinear muscle properties are responsible for a significant reduction in energy expenditure and that they can also contribute to the wave speed difference. This work is a key step in a general program to build integrated “whole animal” models of locomotion and other behaviors that will also allow us to incorporate proprioceptive and exteroceptive neural feedback. Such integrated models can contribute both to our understanding of how living systems work and to the further development of robot systems.

## Introduction

Most fish swim by rhythmically passing neural waves of muscle activation from head to tail, alternating left and right. This yields travelling waves of local muscle shortening, which in turn produce travelling waves of body curvature. These mechanical waves interact with the water, developing reactive thrust that pushes the animal forward. Breder [1] divided this type of swimming into two classes, depending on the proportion of the body undergoing undulations. In the *anguilliform* mode, as exhibited by, e.g. lampreys and eels, most or all of the body is flexible and participates in the propulsive movement. In *carangiform* swimming, as exhibited by, e.g. mackerel, the amplitude of lateral motion is concentrated near the tail. See [2] for an overview of animal locomotion, and [3]–[5] for vertebrate swimming in particular.

**Figure 1:**
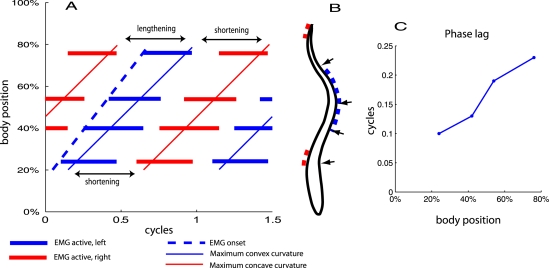
Relative timing of activation and movement. (A) Passage toward the tail of the waves of activation (EMG) and curvature. Speed of activation wave (gradient of dotted line), 1.0 body lengths/cycle. Solid lines show curvature toward left side; speed of mechanical wave (gradient of solid line), 0.72 body lengths/cycle. Arrows indicate time periods over which the muscle on the left side is lengthening and shortening. Abscissa, time (cycles); ordinate, position on body. (B) Lamprey outline from above. Arrows show electrode placement on left side only; dashed lines show active region on each side at approx 0.6 cycles in panel A; (C) Phase delay (fraction of cycle) from onset of activation to onset of shortening (time of maximum convex curvature), plotted against position on body. Data replotted from [6],[7].

At any point on the body, rhythmic cycles of muscle activation alternate with silence, causing cycles of muscle shortening and lengthening (see Figure 1A). However, in all species which have been studied [8] except the leopard shark [9], delays between the onsets of activation and of shortening increase along the body from head to tail (see Figure 1C), i.e., the wave of shortening travels more slowly than the wave of activation. In consequence, near the tail the greater portion of the activation phase occurs during muscle lengthening, giving rise to negative work during part of the cycle. There are a number of possible functions assigned to this change in timing (e.g., providing stiffness as the tail moves laterally through the water, thereby contributing to power transmission, or tuning the resonant body frequency to match tailbeats [10]), but the mechanism or mechanisms responsible for it are not known [11]. In this paper, we throw light on this phenomenon.

Previous computational models of anguilliform swimming have incorporated the known timing of muscle activation within a mechanical representation of the body and water [12],[13], resulting in a travelling mechanical wave. In [13] no phase delay was seen between the waves of activation and curvature, and in [12], none was reported. However, both models assumed specific scalings of muscle density with body location, and that muscle force was simply proportional to activation. In reality, the force developed by activated muscle takes time to develop. Furthermore, because of the changing relative timing of activation and curvature, the patterns of muscle length and velocity vary significantly along the body length. This results in changing patterns in the developed muscle force, and such variation is further complicated by the body taper.

In the present study we investigate this phenomenon by incorporating a revised version of a kinetic muscle force model, originally due to Williams et al. [14], in the continuum mechanical model for anguilliform swimming of [13]. The resulting integrated neuromechanical system models the swimmer as an elastic rod with time-dependent preferred curvature arising from interactions of muscles with the body configuration. The model's modular structure—coupled sets of differential equations—allows us to selectively “lesion” it to probe the sources of its collective behavior. We find that the wave speed difference results primarily from the body's tapered geometry and passive viscoelastic damping, and that it does not require prioprioceptive sensory feedback. Depending on force density, the nonlinear dependence of force on muscle length and shortening velocity can also contribute to the wave speed difference, although it is not *necessary* for it. In a preliminary study, however, we find that length and velocity dependence can reduce the mechanical work output during swimming. When further coupled with a central pattern generator and motoneurons, this integrated muscle-body-enviroment model will also allow us to examine proprioceptive feedback, cf. [15].

This paper is organized as follows. In the methods section we review the equations of motion of the actuated rod and the fluid loading model. We show that the discretized rod equations are equivalent to equations describing a chain of interconnected links. This allows us to relate torques at the joints, and the forces responsible for them, to the preferred curvature and elastic properties of the rod. The model for muscle forces is developed in the penultimate subsection and in the final subsection we combine the muscle and body models to produce an integrated computational model. Simulations of the model are presented in Results and a discussion ensues in the concluding section, in which some larger implications of the work are noted.

## Methods

###  An Integrated Model and Its Computational Realization

We model the swimmer's body as an isotropic, inextensible, unshearable, viscoelastic rod that obeys a linear constitutive relation and is subject to hydrodynamic body forces. We assume that passive material properties such as density and bending stiffness remain constant in time, but allow them to vary along the rod. We endow the rod with a time-dependent *preferred curvature* in the form of a traveling wave, representing muscular activations. We adopt the conventions of [16],[17], and use an elliptical cross section to compute hydrodynamic reaction forces, although we restrict to planar motions, since lampreys and eels in “normal” steady swimming flex their bodies primarily in the horizontal plane [18],[19]. The calcium kinetics and muscle force model, which produces the preferred curvature, is described in the penultimate subsection and the integrated model is summarized in the final subsection of this section. The material of the first three subsections below is drawn from [13], to which the reader should refer for further detail, and where the numerical method and validation tests are also described.

### A Continuum Description of the Actuated Rod

**Figure 2:**
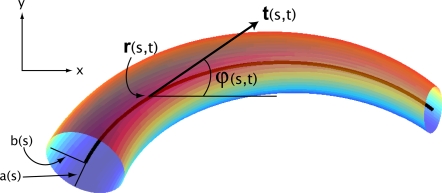
A viscoelastic rod with elliptical cross-sections and variable semi-axes undergoes bending motions in the (*x*,*y*) inertial coordinate plane; *s* denotes arclength along the body centerline. (Figure modified from Figure 1 in [13]).

The independent variable *s*∈[0,*l*] denotes arc-length along the rod, and a *configuration* of the rod is given at each time *t* by the space curve *s* ↦ **r**(*s*,*t*) = (*x*(*s*,*t*),*y*(*s*,*t*)) describing its centerline in the inertial (*x*,*y*)-plane. Derivatives with respect to *s* and *t* will be denoted by subscripts. The inextensibility condition |∂**r**/∂*s*| = 1, can be written in terms of the angle *ϕ* between the tangent to the curve **t** = ∂**r**/∂*s* and the inertial *x*-axis:
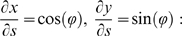
(1)see Figure 2. The normal to **r** is then given by **n** = (−sin *ϕ*, cos *ϕ*). Each element of the rod is subject to contact forces **f** = (*f*,*g*), a contact moment *M*, and body forces W = (*W_x_*,*W_y_*) per unit length, vector components again being referred to the inertial frame. The contact forces and moment are those exerted on the region (*s*,*s*+*ds*) by [0,*s*), which maintain the inextensibility constraint, and the body forces arise from interactions with the fluid environment.

Balance of linear and angular momenta yields the equations of motion (cf. [17],[20]):
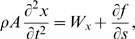
(2)

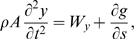
(3)

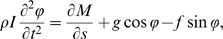
(4)where *ρ* is the volumetric material density and *A* and *I* the cross-sectional area and moment of inertia of the rod. For an elliptical cross-section with semi-axes *a* and *b*, as in Figure 2, *A* = π*ab* and the moment of inertia for motions in the (*x*,*y*)-plane is 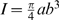. We assume that *ρ* is constant, but allow *A* = *A*(*s*), *I* = *I*(*s*) to vary (both remaining strictly positive); specifically, we will study a tapered elliptical cross section based on lamprey body geometry.

In [13] the activation of the rod was determined by an externally-specified function *κ*(*s*,*t*), representing its *intrinsic* or *preferred curvature*. The muscle model developed later in this section effectively replaces *κ* with a function that depends on neural activation and the local curvature and its rate of change, but we retain the usual linear constitutive relation [20] so that moments are proportional to departures from preferred curvature:
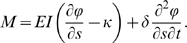
(5)Here *E*>0 and *δ*≥0 are the Young's modulus and viscoelastic damping coefficient and the flexural rigidity *EI*, with SI units N m^2^, determines the overall stiffness. The equations of motion (Equations 2–4), the constraints (Equation 1), and the constitutive relation (Equation 5), along with specified body forces and suitable boundary and initial conditions, form a closed system of evolution equations. Natural boundary conditions for free swimming are that contact forces and moments vanish at the head and tail: *M* = *f* = *g* = 0 at *s* = 0,*l*.

### Approximation of Hydrodynamic Reaction Forces

In swimming the local body forces are due to hydrodynamic reactions that depend on the global velocity field of the fluid relative to the body. To avoid the complexity and computational expense of solving coupled rod and Navier-Stokes equations, we adopt the model of G. I. Taylor [21] in which **W**(*s*,*t*) depends only on the local relative velocity. This approximation accurately predicts forces on a straight rod in steady flow, but fails to capture unsteady effects including vortex shedding, which are undoubtedly important in swimming propulsion [22],[23]. We believe that it suffices as a first approximation for the present purpose, since we are mainly concerned with the interaction of muscle forces and configuration dynamics. Unlike the Kirchhoff and Lighthill theories [24],[25], we neglect added mass effects. See [13] for further discussion.

Taylor models the force on a rod of radius *a* due to perpendicular flow of fluid of density *ρ_f_* and dynamic viscosity *μ* with speed *v* as
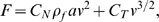
(6)where the drag coefficient *C_N_* varies between 0.9 and 1.1 for Reynolds numbers 20<*R*<10^5^, and *C_T_* is closely approximated by 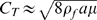 in the range 10<*R*<10^5^, cf. Figure 1 of [21]. Drag forces for smooth oblique cylinders can be decomposed into normal and tangential components in terms of the normal and tangential velocities *v*_⊥_ and *v*_∥_ at (*s*,*t*) as:
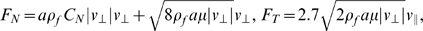
(7) and the body forces are given by
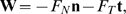
(8)where **n** and **t** denote the normal and tangential unit vectors to the rod's centerline at *s*.

In calculating **W**, we consider only the height 2*a* of the rod, assuming that fluid reaction forces are equal to those on a cylinder of radius *a*, although the constant *C_N_* does change slightly for elliptical rods. Further, we set *C_N_* = 1, since Reynolds numbers for lampreys and eels lie well within the range 20<*Re*<10^5^; for example, in their work on the eel *Anguilla rostrata*, Tytell and Lauder cite *Re* = 60,000 based on body length *l* = 20 cm for a specimen swimming at 1.4*l*/s. [22], and speeds reported in [23] range from 0.5 to 2 body lengths per second. In terms of Taylor's body-diameter-based Reynolds number, this corresponds to *R*≈2000–8000.

### Discretization of the Actuated Rod

**Figure 3:**
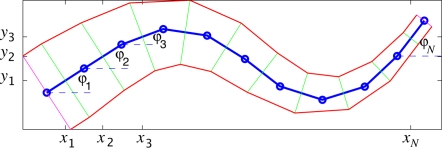
Representation of the swimmer as a chain of interconnected links.

We discretize the rod equations with spatial step size *h* = *l*/*N* in the arclength variable *s*, letting *x_i_*(*t*) = *x*(*ih*,*t*), *i* = 0, …, *N*, and similarly for the other field variables *y_i_*,*ϕ_i_* and parameters *A_i_*,*I_i_*: see Figure 3. The inextensibility constraints in Equation 1 are approximated by
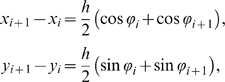
(9) and Equations 2–4 are approximated by the ordinary differential equations (ODEs):
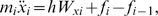
(10)

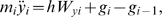
(11)

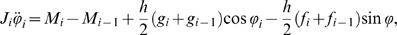
(12)where *m_i_* = *ρA_i_h* and *J_i_* = *ρI_i_h*. The constitutive relation in Equation 5 becomes:
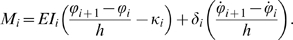
(13)The force and moment free boundary conditions *M* = *f* = *g* = 0 at *s* = 0,*l* become:
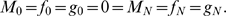
(14)


**Figure 4:**
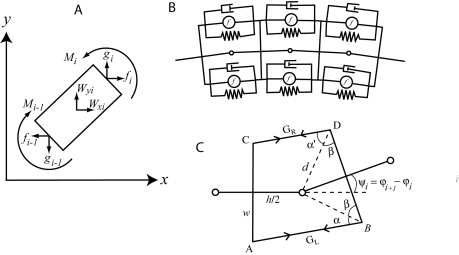
Forces and moments acting on link *i* (A), bending moments are determined by muscles on both sides of the body modeled by springs and dashpots with additional active elements (B), and forces and moments associated with a single joint (C).

The finite-difference discretization of Equations 10–13 is closely related to representions of the body as a planar chain of rigid links subject to forces and moments. In modeling lamprey Bowtell and Williams [26],[27] take a chain of *N* massless rigid rods each of length *h*, with mass *m_i_* at each pivot and at both free ends. The pivots are actuated by passive springs, dashpots, and active force generators. Ekeberg [12],[28] adopts a similar configuration but in place of time-dependent force generators, the spring constants vary with time, and instead of point masses at the pivots, the center of mass of each link is placed at its midpoint. Here we adopt the mass distribution of [12], and include active muscle elements, to be described in succeeding subsections, in the force-generating components. The configuration of the *i*th link is described by its midpoint (*x_i_*,*y_i_*) and the angle *ϕ_i_* between its centerline and the inertial basis vector **ê** *_x_* (Figure 3). Equaions 9 then express the constraint that links remain connected at the joints. Letting (*f_i_*,*g_i_*) and *M_i_* denote the components of contact force and the torque at the joint connecting link *i* to link *i*+1 and (*hW_xi_*,*hW_yi_*) be the body force acting on the midpoint of link *i* (Figure 4a), balances of linear and angular momenta yield Equations 10–12 above with mass *m_i_* = *ρA_i_h* and moment of inertia 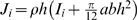 of the *i*th link. The discrepancy between the discretized rod equations and the equations for the chain of *N* pivoted rods thus consists only in the 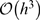 terms in the moments of inertia, and the two models coincide in the limit *h* → 0. We employ the exact formula above for the moments of inertia *J_i_* in all the calculations below, although the approximation *J_i_* = *ρhI_i_* yields results (not shown) that are nearly identical, even for quite large values of *h*≈1.

As shown in section 4.3.4 of [13], for the large segment numbers 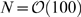 typical of eels and lampreys, the behaviors of the discrete and continuum models are very close. Additionally, the discretization reveals how activation determines preferred curvature *κ*(*s*,*t*) and affects bending stiffness *EI* of the continuum model. As in [26], the joint connecting each pair of links of length *h* is actuated by a pair of spring-dashpot-actuators in parallel, with spring constant *ν* and damping coefficient *γ*, anchored to arms of length *w* that project normally from the links' midpoints (Figure 4b). These arms represent myosepta, the connective tissue layers to which the muscle fibres connect. The linear springs and dashpots represent passive tissue viscoelasticity, and the actuators generate prescribed contractile muscle forces *f_Li_* and *f_Ri_* on the right and left sides of the body respectively. Suppressing the dependence on *i* and denoting the relative extensions 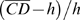 and 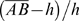 of the spring-dashpot-actuators as Δ*_R_* and Δ*_L_* (Figure 4c), the total forces on the right and left sides may be written
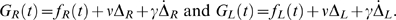
(15)Since the relative extensions are dimensionless, stiffness *ν* and damping *γ* have the units N and N s. respectively. The springs are in tension (and hence generating contractile forces) when Δ*_R_*, Δ*_L_*>0. The forces are applied at a distance *w* from the centerline of the rod, so elementary trignometry gives:
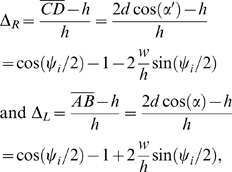
(16)where *ψ_i_* = *ϕ_i_*_+1_−*ϕ_i_* is the angle between neighboring links and 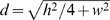. Finally, computing the moment arms *L_R_*,*L_L_* to the joint along normals from the lines *AB* and *CD* on which the forces act (Figure 4c):
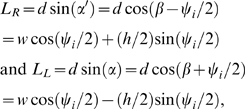
(17) we find that, for small angles *ψ_i_*, the resulting torque at joint *i* is given by
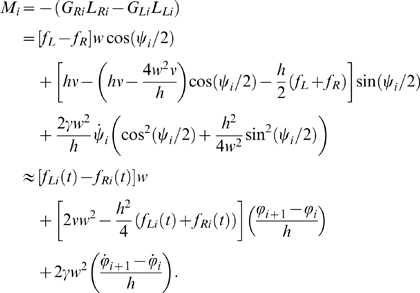
(18)


Comparing the linearized moment in Equation 18 in the limit *h* → 0 with the discretized constitutive relation in Equation 13 we see that the link and discretized rod models coincide if the stiffness *EI_i_*, intrinsic curvature *κ_i_* and viscoelastic damping *δ* are interpreted as follows:
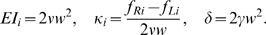
(19)


We propose that the stiffness *ν* and damping *γ* are proportional to cross-sectional area *A*(*s*). Thus we set
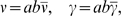
(20)so that the stiffness 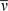 and damping 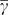 have units N/m^2^ and N s./m^2^ respectively. To approximate a uniform distribution of the muscle, we set *w* = *b*/2, where *b* is the half-width of the body. Equations 19 now become
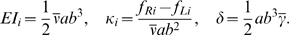
(21)In particular, using *I* = *πab* ^3^/4 we can write Young's modulus in terms of the spring stiffness as 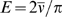. One of the questions we address is the influence of force density as a function of arclength. We take up this question after a discussion of force generation in muscle fibers.

### Muscle Activation and Force Generation

Recordings such as those of [29] show that waves of motoneuronal activity consisting of bursts of closely-spaced action potentials (APs), separated by near-silent interburst periods, travel the length of the lamprey spinal cord (see Figure 1A and 1B). The waves are generated spontaneously by a distributed central pattern generator (CPG) within the spinal cord [30], which has been modelled as a chain of coupled oscillators [31]–[33]. The waves are in antiphase contralaterally and maintain approximately constant duty cycles (burst/cycle period ratios) and segment-to-segment ipsilateral phase lags, regardless of overall frequency. This activity pattern is transmitted via nerves that enter the myotomes through the ventral roots [34], producing muscle activation with similar phasing, evident in electromyograms (EMGs) [7]. Each myotome corresponds to a segment of the spinal cord.

Bundles of myofibrils make up the muscle fibres within the myotomes. The AP bursts cause calcium release from the sarcoplasmatic reticulum (SR) that surrounds the myofibrils and is encircled by T-tubuli at repeated intervals. The resulting muscle contraction occurs in three phases. (i) A motoneuronal AP arrives at the neuromuscular junction, producing an AP at the motor end plate which spreads along the surface and T-tubular membranes of the muscle fiber. (ii) This depolarization opens gates in the SR and releases Ca^2+^ ions into the muscle protein filaments. (iii) Ca^2+^ causes conformational changes in the thick filaments which form cross-bridges to the thin filaments; a subsequent conformational change then develops a force tending to slide the thin filaments over the thick ones [35], shortening the muscle (unless overcome by opposing force via the muscle attachments). This is followed by resequestering of Ca^2+^ by the SR, resulting in relaxation of the muscle. The force developed during muscle activation is dependent upon both the length of the muscle and the velocity of its shortening [36]. Traditionally, shortening is taken as positive, but here we use the opposite convention, referring to the time derivative of muscle length as *velocity*, which is negative for shortening.

**Figure 5:**
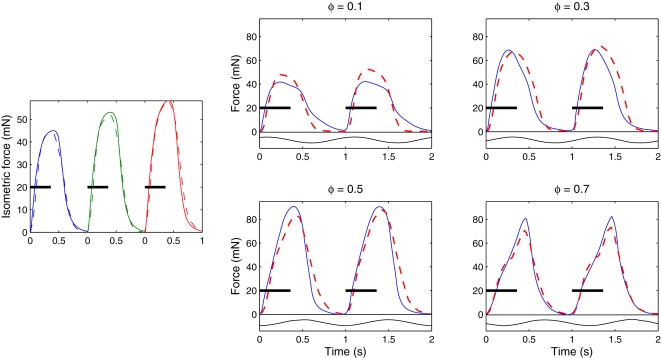
Least squares fit of model to isometric data at three muscle lengths (left), and sinusoidal forcing data with predictions from isometric data fit (right). Tetanic stimuli applied periodically for 0.36 s, while length of preparation varies sinusoidally with various phase offsets *ϕ*. The sine waves show the length of the preparation as a function of time. Solid lines, simulation; dashed lines, data.

**Figure 6:**
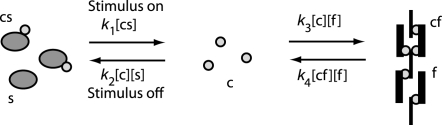
The model of calcium kinetics. *c*, free calcium ion; *s*, unbound SR calcium-binding sites; *cs*, calcium-bound SR sites; *f*, unbound contractile filament calcium-binding sites; *cf*, calcium-bound filament sites; *k*_1_–*k*_4_, rate constants of binding and release.

To describe the forces *f_R_*(*t*) and *f_L_*(*t*) in Equations 15, 18, and 19, we adapt the model developed by Williams et al., who carried out experiments on portions of single myotomes of lamprey muscle [14]. Intermittent tetanic stimulation was applied during isometric and constant-velocity movements, and analysis and modelling of the resulting force trajectories were used to predict the trajectories recorded during applied sinusoidal movement. Experimental data are reproduced in Figure 5 below (for details of experimental protocol, see [14]). We follow a modified form of the simple kinetic model used in that study, including calcium ions, SR sites and contractile filaments (CF). The rates at which calcium ions are bound and released approximately follows the principle of mass action (see Figure 6). For example, the rate of binding of calcium ions to the CF is proportional to the product of concentrations of free calcium ions and unbound filaments, with rate constant *k*_3_. The resulting equations for the kinetics of the calcium, sarcoplasmic reticulum sites and bound filaments are as follows:
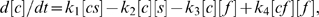
(22)

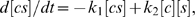
(23)

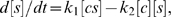
(24)

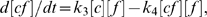
(25)

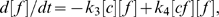
(26)where brackets denote concentrations of the relevant quantity. When the muscle is activated, *k*_1_>0 and *k*_2_ = 0; in the absence of activation *k*_1_ = 0 and *k*_2_>0.

We assume that the total number of calcium ions, SR binding sites and filament binding sites per liter remain constant so that [*cs*]+[*c*]+[*cf*] = *C_T_*, [*cs*]+[*s*] = *S_T_*, and [*cf*]+[*f*] = *F_T_*. This allows us to reduce the five Equations 22–26 to a system of two in [*c*] and [*cf*]. We further scale by the number of filament sites *F_T_*, writing *Caf* = [*cf*/*F_T_*], *Ca* = [*c*]/*F_T_* and introducing the new constants *C* = *C_T_*/*F_T_* and *S* = *S_T_*/*F_T_*. Since the number of bound filament sites cannot exceed *F_T_*, *Caf*≤1, *Ca*≤*C*, and *Caf* = 1 when all of the filaments are bound. Although appropriate values for *C* and *S* are not known, general knowledge of skeletal muscle indicates that *C* is large enough for the filament binding sites to be saturated during tetanic stimulation and that *S* is large enough to reduce free calcium to a negligible amount during rest. We obtain similar data fits over a range of values for these constants, so we arbitrarily set *C* = 2 and *S* = 6. Thus twice as much calcium is available than is necessary to bind all of the filaments and thrice as many binding sites are available in the SR than are required to bind all the calcium.

Following Hill [37], each myotome is modeled as a contractile element (CE) in series with an elastic element (SE). (The Hill model includes a second elastic element in parallel [38], but for our purposes this can be included in the linear spring of Figure 4b.) Because they are in series, the CE and SE experience equal forces at steady-state. We begin by describing them separately, as a force *P* exerted by the SE, and a force *P_c_* developed by the active element CE.

The SE is modelled as a linear spring and hence *P* is proportional to the length *l_s_* of this element minus its resting length *l_s_*_0_: *P* = *μ_s_*(*l_s_*−*l_s_*_0_). This force is never negative. The total length *L* of the segment is the sum of *l_s_* and the length *l_c_* of the contractile element. The length and velocity *v_c_* = *l* ˙*_c_* of the contractile element are therefore given in terms of the length and velocity *V* = *L* ˙ of the segment and the force *P* as follows:
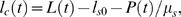
(27)

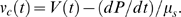
(28)


We assume that the the force *P_c_* exerted by the contractile element can be described by independent multiplicative factors of its length *l_c_* and velocity *v_c_*,
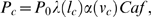
(29)where the constant *P*_0_ is the force exerted in isometric tetanic contraction (*Caf* = 1) at the optimum length *l_c_*_0_. The functions *λ*(*l_c_*) and *α*(*v_c_*) are estimated from force measurements (described below), from which we obtain a piecewise linear function for *α* and a quadratic for *λ*:
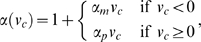
(30)

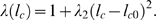
(31)We additionally restrict these functions such that 0≤*α*(*v_c_*)≤*α*_max_ and 0≤*λ*(*l_c_*)≤1. The fact that *α_p_*>*α_m_*>0 (see Table 1) reflects the ability of muscle fibers to exert progressively greater forces during lengthening than in shortening.[Table pcbi-1000157-t002]

**Table 1:** Muscle model parameters used in simulations.

From data				From best fits		
Parameter	Value	Parameter	Value	Parameter	Isometric	Sine
*μ_s_*	600 mN/mm	*α_m_*	0.40 s/mm	*k*_1_	9.6 s^−1^	10 s^−1^
*l_is_*	2.7 mm	*α_p_*	1.33 s/mm	*k*_2_	5.9 s^−1^	12 s^−1^
*l_s_*_0_	0.234 mm	*α*_max_	1.8	*k*_3_	65 s^−1^	49 s^−1^
*λ*_2_	−2.23 mm^−2^	*k*_5_	100 s^−1^	*k*_4_	45 s^−1^	40 s^−1^
*l_c_*_0_	2.6 mm			*P*_0_	60.86 mN/mm^2^	72 mN/mm^2^

**Table 2 pcbi-1000157-t002:** List of major symbols.

Symbol	Definition	Symbol	Definition
**r**	Centerline of rod	*ϕ*	Angle between tangent and *x*-axis
*a*	Semi-major axis (height) of rod	*b*	Semi-minor axis (width) of rod
*ρ*	Volumetric density of rod	*A*	Area of cross-section
*I*	Moment of inertia of cross-section	*M*	Moment
**W** = (*W_x_*,*W_y_*)	Body (fluid) forces	*E*	Young's modulus
*δ*	Viscoelastic damping	*κ*	Preferred curvature
*μ*	Dynamic viscosity	*ρ_f_*	Fluid density
*G_Ri_*_,*Li*_	Total force on right/left of joint *i*	*f_Ri_*_,*Li*_	Active contractile force on right/left of joint *i*
*ν*	Spring constant	*γ*	(Spring) damping coefficient
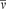	Scaled spring constant (*ν*/*ab*)	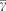	Scaled damping coefficient (*γ*/*ab*)
*P_Ri_*_,*Li*_	Force per unit area of muscle on right/left of joint *i*	*w*	Distance from centerline at which forces are applied
*ζ*	Force density	*L_Ri_*_,*Li*_	Length of muscle on right/left side of joint *i*
*V_Ri_*_,*Li*_	Velocity of muscle on right/left side of joint *i*		

If we set *P_c_* = *P*, the calculation suffers from instability, and in reality the stretch of the SE due to activation of the CE is not instantaneous. We therefore model the transfer of force from the CE to the SE by simple linear kinetics:
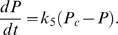
(32)


Combining Equations 22–32 and using the three conserved quantities *C_T_*, *S_T_*, and *F_T_*, we obtain three ODEs for the concentrations of free calcium, bound calcium and the force exerted by the preparation:
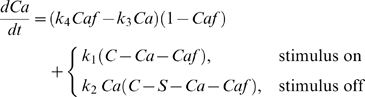
(33)

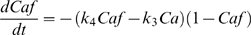
(34)

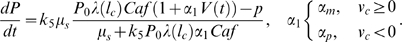
(35)


The parameters of the model are determined from analysis of the data of [14], as follows. *μ_s_* and *l_s_*_0_ are determined from quick-release experiments [37]. The maximum values of force *P*_0_ in the three isometric experiments (Figure 5) are used in Equations 27, 29, and 31 to determine the values of *λ*_2_ and *l_c_*_0_. The results of constant-velocity ramp experiments are then used with Equations 27–31 and the parameters *λ*_2_ and *l_c_*_0_ to determine *α_m_* and *α_p_*. The limiting value of *α*_max_ was not determined in [14], so *α*_max_ is taken from results in dogfish [39]. In practice, results vary little over a range of values for *α*_max_.

We set the time constant *k*_5_ = 100 s^−1^, so that *P_c_* closely tracks *P*. The remaining time constants *k*_1_, *k*_2_, *k*_3_, and *k*_4_ are found by fitting force trajectories from the experimental data, using the least-squares curve-fitting facilities in the software *XPPAUT* devised by G. Bard Ermentrout and available at http://www.pitt.edu/phase/.

The parameters *k*_1_–*k*_4_ are fit in two different ways. The *isometric fit* follows the approach in [14] by using only data from the isometric experiments at the three lengths *L* = 2.7 and 2.7±0.125. The main aim of [14] was to show that a model based on isometric and constant-velocity experiments could be used to approximately predict forces that occur during swimming, even though it excludes known properties such as the observation that the length-tension and force-velocity relationships change during muscle activation and relaxation [36]. Such secondary features cause discrepancies between the predictions and the data seen in the sinusoidal traces of Figure 5, but the model nonetheless produces forces during sinusoidal movement that capture the overall behavior well.

**Figure 7:**
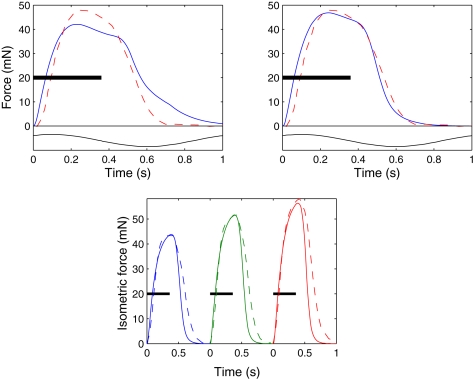
Fit of model to sinusoidal forcing data. The upper left panel is the same as that in Figure 5 for phase offset *ϕ* = 0.1; the upper right panel shows the model behavior under the same conditions but with rate parameters *k*_1_–*k*_4_ fit to the sinusoidal data. Lower panel shows the model behaviour under isometric conditions with these parameters. Solid lines, simulation; dashed lines, data.

The present study demands our best estimate of force development during swimming, and for this reason we have made a second, *dynamic fit* of the time constants *k*_1_–*k*_4_ based not on isometric data but on muscle force data during sinusoidal movement at 1 Hz. To best match swimming behavior, we chose the experiment with a delay of 0.1 from onset of stimulation to onset of shortening (cf. Figure 1), and as the upper panels of Figure 7 show, the resulting force trace is much closer to the data than the fit to isometric data. The discrepancy between the isometric data and the prediction using these parameters is primarily in the repolarisation phase (Figure 7, lower panel), reflecting the model's inadequacy during this phase of the force trajectory. Values for both fits, along with the other muscle parameters, are given in Table 1. The most striking difference is in the rate constant *k*_2_ (uptake of free Ca^2+^ by the SR), which doubles. Using this, the dynamic fit captures the rapid force decay seen in the sinusoidal data at low phase delays.

Sinusoidal forcing data were only available at 1 Hz [14] and in most of the simulations described below we retain this frequency, but we also briefly investigate swimming behavior at 2 Hz. The muscle parameters are listed in Table 1. It is worth noting that neither set of time constants is unique: in both cases it was possible to find more than one set of time constants that gave a good fit, by starting from different initial guesses. The primary goal of this study is not to discover accurate parameters, but to find a good prediction of muscle behaviour for use in our neuromechanical model.

### The Integrated Model

Muscle dynamics is incorporated into the discretized rod model as follows. The forces *P_Ri_* and *P_Li_* generated by the right and left myotomes associated with the *i*th link are modeled by two sets of the three Equations 33–35, with maximal force *P*_0_ scaled by cross-sectional body area at that location. Thus, if the entire body length is actuated, 6(*N*−1) first order ODEs describe the muscle forces in the *N*-link chain, and with the 3*N* second order ODEs in Equations 10–12 they jointly determine the body dynamics. Unlike in the simplified model of [13], the time course of force development now depends on the proportion of activated filaments (*Caf*) and on the lengths and velocities of the muscle fibers, via appropriately scaled versions of Equations 27–32. At joint *i* the lengths and velocities are
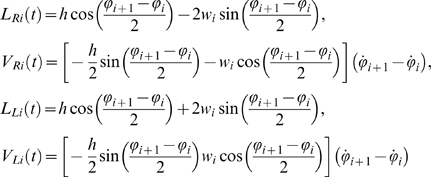
(36)(see Figure 4 and the discussion in the preceding subsections). Equations 36 provide the explicit coupling between the muscle and body equations. As in [13] the preferred curvature at joint *i* is given by 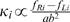, and the force at each segment is given as a scaled multiple of the force *P_R_*_,*L*_ of the fibers on either side of the joint. Since the number of fibers typically depends on cross-sectional area, our first approach was to take *f_R_*_,*L*_∝*abP_R_*_,*L*_, giving a preferred curvature *κ_i_*∝(*P_R_*−*P_L_*)/*b*, but simulations with such a relation exhibited much greater motions toward the tail than those seen in the swimming animal. After extensive simulations with various scalings (not shown), we found that scaling the preferred curvature as *κ_i_*∝*b* ^2^(*P_R_*−*P_L_*) and the stiffness as *EI*∝*ab* ^2^ provides the best qualitative match to behavior. This suggests that the Young's modulus, and hence 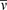, *increases* along the length of the body, while not only the magnitude, but also the density of muscle forces *decreases*. The former is consistent with the fact that the notochord takes up a proportionally greater portion of the cross-section of the animal toward the tail. This scaling thus corresponds to 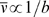, and *f_R_*_,*L*_∝*ab* ^3^ *P_R_*_,*L*_, cf. the middle equation of Equation 21.

In the experiments described above the stimulus applied was tetanic, which does not occur normally. We assume that during swimming the muscle is stimulated in such a manner that it can be scaled linearly with respect to the tetanic stimulus. We thus scale the forces with a constant *ζ* that is chosen ad hoc, so that
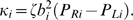
(37)Equation 37 completes the loop, so that upon imposing a traveling wave of activation which releases calcium by setting *k*_1_ and *k*_2_ of Equation 33 on and off in a piecewise constant square wave (approximating the EMG recordings [29]), we obtain a closed system of ODEs.

## Results

We now explore the behavior of the discretized model actuated by forces generated by segmental muscles. It is important to note that the entire body length of a fish is usually not equipped with swimming muscles. In lamprey the head and part of the gill region lack such muscles, and we shall henceforth assume they occupy 1/10 of the total body length, and that their passive material properties are the same as those of the rest of the body. We shall refer to the remaining 9/10 of the body that is capable of activation [40] as the *activation region*.

Only a fraction of the myotomes are activated on either side of the body at each instant, in a region that travels from head to tail during normal swimming. The mean temporal duration of activation at a given location is ≈0.36 of the mean cycle duration [7] (see Figure 1). This defines the square wave referred to above, and implies that the activated portion on either side also has length 0.36 times the activation wavelength. We will generally assume that the activation wavelength is one body length [6] (i.e., greater than the length of the activation region). Unless otherwise stated, in the simulations reported below we apply a stimulation rate of 1 Hz, and the activation wave thus travels down the body at speed of 1 body length/s.

The value of the Young's modulus *E* (or 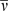, cf. Equation 19) for the lamprey is not known with any precision. However, the studies in [10] suggest a value of *E*≈0.1 MPa for the eel, and the lamprey's passive stiffness is thought to be much smaller. Indeed, the stiffness of an anesthetized lamprey is so low that is difficult to measure, but preliminary studies suggest that values in the range 10^−3^−10^−2^ MPa are not unreasonable [41]. Other parameters for which we have no firm lamprey data are the overall scale of the muscle force *ζ* and the damping 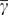. The values specified here are selected based on extensive exploratory simulations and the studies of passive elastic and geometric properties in [13].

We take a tapered rod of length *l* = 21 cm with constant height 2*a* = 2 cm, to account for the dorsal and anal fins, and width 2*b*(*s*) given by *b*(*s*) = 1−(4/5)(*s*/*l*) cm. Unless otherwise stated, the following body parameter values are used throughout this section: Young's Modulus *E* = 10^−3^ MPa, damping 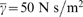, and force density *ζ* = 0.05 N/m^3^. Fluid density and viscosity are *ρ_f_* = 1 g/cm^3^ and *μ* = 10^−3^ Pa·s – the values for fresh water – and the body is discretized into *N* = 21 links. We refer to these as the standard or control parameters.

We use a numerical method adapted from that of [13], the appendix of which contains a detailed description. The main difference is incorporation of two sets of the muscle force Equations 33–35 at each joint. These are in turn coupled with the rod equations through the preferred curvature *κ*, explicitly via the length and velocity of the muscle as described by Equations 36 above. The method employs discrete versions of the integrated constraint equations in Equation 1 that express link positions in terms of that of the head (*x*_1_,*y*_1_) and the link angles *ϕ_i_*, thus guaranteeing that the inextensibility constraint is precisely satisfied for the discrete system in Equations 9–13 and eliminating the need to solve ODEs for (*x_i_*,*y_i_*) *i* = 2, …, *N*. Since the head region lacks activation, the number of ODEs required to describe muscle forces reduces to 6(*N*−1)×(9/10) = 108 in the present case.

### Simulations of Swimming and Body Shapes

**Figure 8:**
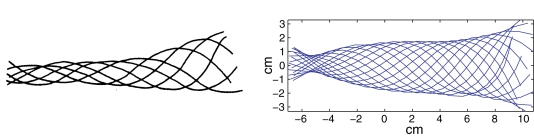
Tracings from a swimming lamprey, reproduced from [26] (left), and centerline of the actuated rod at various times (right). Head is at left in (A) and (B).

**Figure 9:**
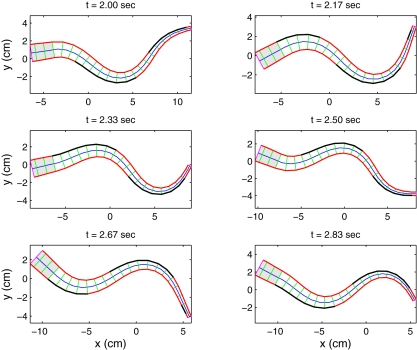
Snapshots of the muscle-actuated swimming rod over one period of activation at a frequency of 1 Hz. Bolded segments on either side indicate muscle activation (*k*_1_>0). Shaded region represents the unactuated head. Note that curvature lags behind the activation wave.

**Figure 10:**
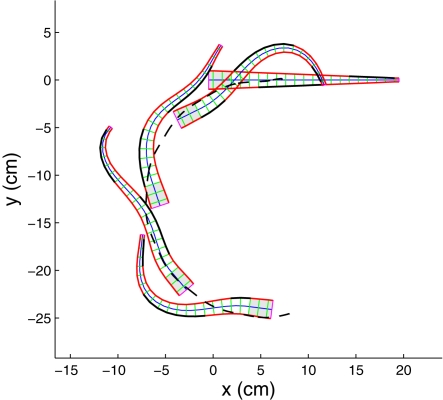
Setting the force density in activated regions on the right side half that on the left causes a steady turn. Dashed curve shows the path traveled by the center of mass.

Simulations readily yield results that are qualitatively similar to real anguilliform swimmers. For example, Figure 8 compares tracings from a film of a lamprey in a swimmill that approximate its body centerline at various times with centerline snapshots from a model simulation. The characteristic swimming behavior is clearly captured, in particular the larger amplitude at the tail end. Figure 9 shows snapshots of the body over one activation cycle. When the force density magnitude *f_R_*_,*L*_ is the same on both sides, the center of mass travels in a nearly straight line, with small lateral oscillations that arise due to slight asymmetries in body shape (see section 4.3.2 of [13]). The mechanical wave travels down the body at a speed of 0.78 body lengths/s, producing a forward swimming speed that rises asymptotically to a value of 0.40 body lengths per second, giving a speed ratio or *slip* of 0.51. Slip values are not available for swimming lamprey, but the expected value for eels swimming at the same speed is 0.66 [23]. It is likely that eels are more efficient swimmers than lampreys, since they do not exhibit the side to side movements of the head seen in lamprey (Figure 8). Turns can be evoked by reducing the magnitude of force density on one side, so that the average of the rod's intrinsic curvature 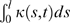 is nonzero: see Figure 10.

### Effects of Muscle, Elastic, and Geometric Properties on Relative Speeds of Activation and Mechanical Waves

We now attempt to determine the mechanism(s) causing the difference in wave speeds of activation (EMG) and curvature. In the simulations of [13], the preferred curvature *κ* = *κ*(*s*−*ct*) was externally prescribed, specifically, as that of a traveling sine wave. The curvature *ϕ_s_* that emerged depended on the passive elastic properties of the rod and the hydrodynamic body forces, but in [13] *κ* itself was independent of the body dynamics and of *ϕ_s_*. In the present model the ODEs in Equations 33–35 couple the preferred curvature to the state of the rod, via the length and contraction speed of muscle fibers (cf. Equation 36) that appear in the functions *α*(*v_c_*) and *λ*(*l_c_*) of Equations 29–31. Hence *κ* now depends on *ϕ_s_*, and we are able to investigate what role this dependence plays in wave propagation.

**Figure 11:**
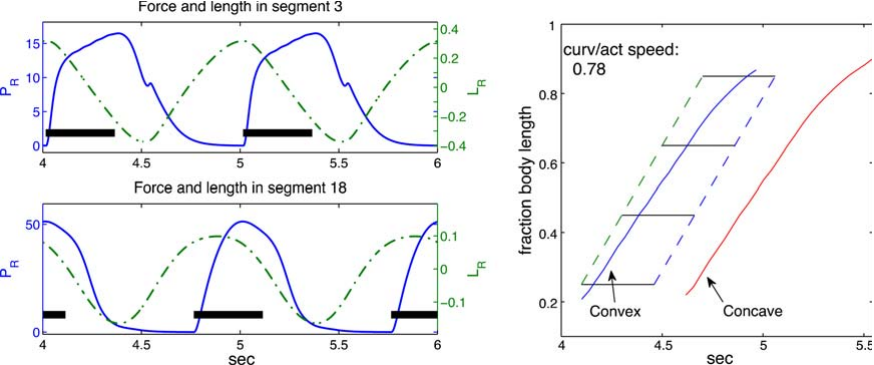
Forces generated by contractile filaments (solid lines, left axes) and resulting length changes (dashed curves, right axes) in the right side of segments 10 (top) and 18 (bottom) of a 21-segment tapered rod (left panels), and passage of activation and curvature waves during one swimming cycle (right panel). (Left) Horizontal bars represent activation period. (Right) Solid curves show locations of maximal concave and convex curvatures. Horizontal lines represent periods during which a segment is activated and dashed lines connect activation onsets and terminations. In body lengths/s, activation wave speed = 1 and mechanical wave speed = 0.78, for a speed ratio of 0.78.

Figure 11 shows the relative timing of activation, muscle force development, and muscle shortening in a typical simulation. Activation waves travel the length of the active region with a frequency of 1 Hz, as in Figure 9. The left panel shows time courses of muscle length and force in two segments on the same side of the body; the right panel shows the relative timing of activation and curvature in the same format as Figure 1. We calculate the average wave speed of the maximal concave and convex curvatures by linear regression, first approximating the angle *ϕ*(*s*) along the rod by a cubic spline interpolant of the joint angles *ϕ_i_*. This yields a continuous function of arclength *s*, from which we estimate the maximal and minimal curvatures. In all cases the mean speeds of convex and concave curvatures agree to 3 decimal places, so we report a single ratio of curvature speed to activation wave speed. As in the lamprey (Figure 1), the mechanical wave is slower than the activation wave, the wave speed ratio being 0.78, within the range of values 0.72±0.07 (SD) observed in lamprey [6].

The wave speed difference could be due to several separate effects, or to some combination of them. Ostensibly, any or all of the following could play roles:

1. Overall force levels.
2. Passive viscoelastic forces.
3. Hydrodynamic reaction forces.
4. Body geometry (taper).
5. Muscle length and velocity dependence.
6. Scaling of muscle forces with body cross section.

**Figure 12:**
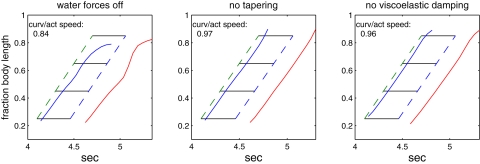
Effects of fluid forces, body taper, and passive damping on the speeds of activation and response waves. Left panel: no fluid forces, W≡0; middle panel: no taper, *b*(*s*)≡1; right panel: no viscoelastic damping, 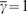.

We now examine these items individually and in combination. First we consider the effect of fluid loading. By setting **W**≡**0**, we remove fluid forces, a situation approximated in the laboratory by stimulating a lamprey to “swim” on a slippery bench [26]. Figure 12 (left panel) shows the results of one such simulation. A difference in wave speeds persists, although in this case the speed of the mechanical wave tends to decrease slightly midbody and then *increase* toward the tail. Eliminating hydrodynamic reaction forces has the effect of further reducing what is already a very small body stiffness. Under the same muscle activations the rod flops around violently.

We varied several parameters, including stiffness, viscoelastic damping, the length of the activated region and wavelength of the activation, and body geometry. As noted above, our value of Young's modulus, *E* = 10^−3^ MPa, is extremely small, but simulations with higher values did not yield realistic results. For example, with *E*≈0.1 MPa and an increase in muscle force density by a factor of 3, the ratio of curvature to activation speeds is 0.9, mean swimming speed increases to 0.5 body lengths per second, but the phase delay between activation and shortening is approximately zero throughout the rod.

We found that two further properties are necessary to create the observed difference in activation and response wave speeds: taper in the body and the presence of viscoelastic damping. Figure 12 (middle panel) shows results of simulations performed on an untapered rod (*b*(*s*)≡1), for which the wave speeds become almost identical. The strongest effect of taper is probably via the reduced muscle cross section, and hence smaller force generation, toward the tail (recall that the “hydrodynamic cross section” used in Equation 7 remains fixed at *a* = 1 cm, and that a wave speed difference persists in the absence of hydrodynamic forces.) The right panel of Figure 12 shows results of simulations performed without viscoelastic damping (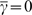). In this case the speed ratio also increases significantly, to 0.96.

**Figure 13:**
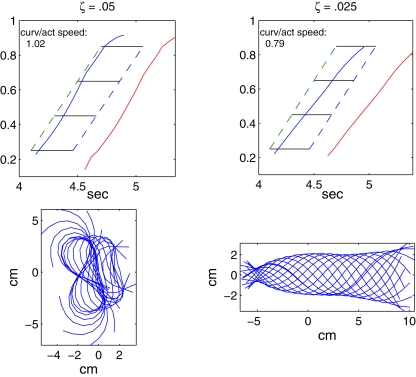
Effects of removal of muscle length and velocity dependence on force generation, the speeds of activation, and curvature and body shapes. In all panels, *λ*(*l_c_*)≡0.86 and *α*(*v_c_*)≡1. Left panel: same overall force density as control; right panel: force density reduced by half to *ζ* = 0.025. Lower panels show snapshots of body centerlines.

Next we consider the effects of eliminating the dependence of muscle force on length and/or velocity, by setting the functions *λ*(*l_c_*) and/or *α*(*v_c_*) of Equations 27 and 28 identically equal to constants. For the former we choose *λ*(*l_c_*)≡0.86, because this is the value of *λ*(*l_c_*) at the middle length (2.7 mm) used in the isometric experiments of Figure 5, and it corresponds to the average length during typical swimming motions [14]. For the latter we take *α*(*v_c_*)≡1, corresponding to zero velocity. Removing both effects and maintaining all other parameter values, including force density *ζ* = 0.05 N/m^3^, we find that the wave speeds are approximately equal, but that mechanical wave amplitudes become unrealistically large (Figure 13, left panels). Upon reducing *ζ* to 0.025 N/m^3^ to achieve reasonable amplitudes, we obtain the result shown in the right panels of Figure 13: i.e., a speed ratio nearly equal to the case in which length and velocity dependence are present, but body motions are now more pronounced near the head, unlike the shapes of Figure 8. The swimming speed also drops slightly from 0.40 to 0.39 body lengths per second, and, as reported in the following subsection, the swimming efficiency is sharply reduced when length and velocity dependence are removed.

**Figure 14:**
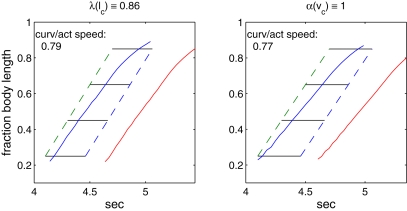
Effects of removal of muscle length and velocity dependence on the wave speeds of activation and response. Left panel: no length dependence; right panel: no velocity dependence. Force density reduced to *ζ* = 0.025 in both cases.

The multiplicative dependence of muscle force on the factors *λ*(*l_c_*) and *α*(*v_c_*) also allows us to separate these effects. In the simulation illustrated in the left panel of Figure 14, we set *λ*(*l_c_*)≡0.86 but retain the function *α*(*v_c_*), thus eliminating length dependence alone. The resulting speed ratio of 0.79 is almost unchanged from the control value for the full model (cf. Figure 11). The right panel of Figure 14 shows the result when only velocity dependence is abolished, by setting *α*(*v_c_*)≡1 and retaining *λ*(*l_c_*). The speed ratio 0f 0.77 is again nearly equal to the control value, although phase lags are reduced over the first half of the body length. Thus, removing *either* length or velocity dependence alone does not significantly affect the difference in wave speeds. In both these cases, and all those to follow, we retained the standard force density *ζ* = 0.05 N/m^3^.

**Figure 15:**
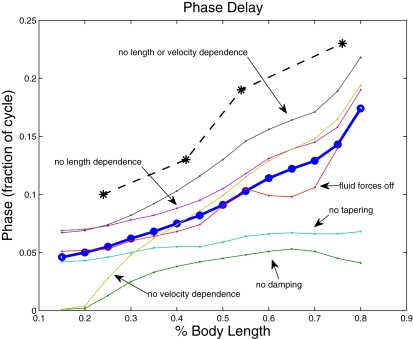
Phase between the start of activation and the beginning of shortening. Stars connected by dashed lines show the phase between the start of EMG activity and the beginning of shortening expressed as fraction of the cycle duration (data from [6], but with phase defined somewhat differently). Thick curve with circles shows data from a simulation when all effects are present; thin curves show results of simulations with effects removed as labeled.

The difference in wave speeds changes the relative timing between muscle activation and shortening as waves travel down the cord, as shown in Figure 1C. The changes in this relationship under all the conditions that we have investigated are illustrated in Figure 15, in which the delay from the beginning of muscle activation to the time of maximal convex curvature (approximately the beginning of shortening) is plotted against body position. The broken line at the top reproduces values from Figure 1C, experiments of [6] showing that the delay increases from 0.10 of a cycle at 24% of the body length to 0.23 at 76% body length. Data from the full control simulation of Figure 11 are shown by the thick blue line. Although the resulting phase lags are smaller than those observed in the animal, the phase gradient is qualitatively correct. Data from the simulations of Figure 12 are also shown, illustrating that with these changes in mechanical properties, the phase lag values are very different from normal. Abolition of length and velocity dependence, as in Figure 13, has little effect, when accompanied by halving the force density. Removing only the velocity dependence, as in Figure 14 (right panel), however, abolishes the phase lag in the most rostral segment.

**Figure 16:**
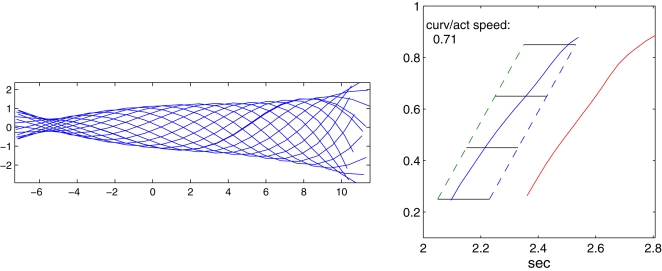
Body centerline snapshots and passage of activation and curvature waves at a swimming frequency of 2 Hz.

The preceding simulations were all done for swimming at 1 Hz, the frequency for which muscle force data is available. Lampreys can of course swim over a range of speeds, by varying both activation levels and frequencies. *Ichthyomyzon unicuspis* has been recorded as swimming at frequencies up to ≈7 Hz., although this probobaly does not represent steady swimming. To verify that our model can accomodate frequency variations, we performed simulations at 2 Hz, keeping all other parameters at their standard values. Figure 16 shows that body shapes and amplitudes remain similar to those at 1 Hz, although the wave speed difference is somewhat magnified, the ratio decreasing to 0.71.

### Swimming Efficiency

As noted above, removing the length and velocity dependence in muscle forces, while simultaneously halving the force density *ζ*, leads to a nearly identical ratio of curvature to activation wave speeds with only a slight reduction in swimming speed. Since nonlinear muscle properties are not *required* to produce the observed speed difference, we were prompted to ask what other differences they make. Here we investigate their effect on swimming efficiency, by comparing the work done by the muscles over a full activation cycle with length and velocity dependence present and absent. We calculate the work done by the muscles on either side of joint *i* by computing the integrals
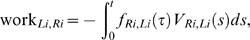
where *f_Ri_*_,*Li*_ and *V_Ri_*_,*Li*_ are the right- and left-hand muscle forces and velocities defined in the last two subsections of Methods (the negative sign is due to our convention that *V_Ri_*_,*Li*_ are lengthening velocities).

**Figure 17:**
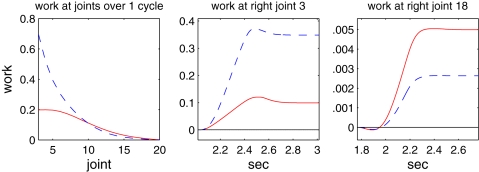
Work done by muscles. Solid and dashed curves respectively show model results with length and velocity dependence present and absent (*λ*(*l_c_*)≡0.86 and α(*v_c_*)≡1). Left: total work done by muscle pairs at each joint over one full cycle; center: work done during one cycle by right hand muscle at joint 3, near head; right: work done by right hand muscle at joint 18, near tail. With length and velocity dependence, substantially less work is done near the head, but slightly more near the tail (note difference in ordinate scales).

The left panel of Figure 17 shows the work done at each joint, illustrating that, in spite of the reduced force density used for the case without length and velocity dependence, 67% more work is done than when length and velocity dependence are included, although there is a slight *reduction* in swimming speed. The difference is largest near the head; the work done near the tail being slightly larger for the latter case. The center and right panels show time courses of work done over one cycle at specific locations in these two regions (joints 3 and 18), with activation beginning at the time on the left axis in both cases. In addition to substantial differences in magnitudes due to reduced muscle cross section near the tail, these panels reveal that negative work is done at the tail in the beginning of the activation phase, while muscles are still lengthening. As we have noted, this may play a role in stiffening the tail as it moves laterally through the water.

Overall, these results suggest that the length and speed dependencies of the muscle fibers may provide a mechanical advantage to the animal in swimming.

## Discussion

This paper is primarily concerned with the role of muscle activation in the production of anguilliform swimming motions: a process that involves multipath coupling among active filaments, passive body tissues, hydrodynamic reaction forces, and proprioceptive and exteroceptive sensory feedback. To better parse this complex coupled system, here we address the influence of “feedforward” neuromechanical coupling alone by means of a mathematical model.

Our model substantially extends previous ones [12],[13],[42] by its inclusion of nonlinear muscle dynamics, which is characterised by known physiological properties with parameters fitted to experimental data. Coupled with appropriate passive viscoelasticity and geometry of the body, this gives rise to a difference in the wave speeds of neural activation and mechanical response, as seen in swimming animals, and the model enables us to investigate the sources of this difference. We find that three factors are primarily responsible for it and for the associated lags between activation and curvature onsets, namely: viscoelastic damping, taper, and the nonlinear dependence of muscle force on length and shortening velocity. The first two factors, which are properties of passive tissues and body geometry, are necessary for the appearance of the wave speed difference. The third factor, nonlinear muscle dynamics, contributes to the values of the changing phase lags, and may also contribute to the efficiency of swimming.

Figure 15 shows that the phase relationship between muscle activation and shortening produced by the model is similar to that seen in the lamprey. Significantly better data fits can be obtained by varying parameters outside the normal ranges, but rather than explore this systematically, we have instead used parameter values that best describe the lamprey.

The present study illustrates the power of integrative mathematical models in revealing biological function, by allowing “experiments” which cannot be done on animals. It partially answers questions posed by Altringham and Ellerby, who conjectured that the progressive phase lag is associated with “change in muscle function along the body [11].” Our study shows that, at least for anguilliform swimmers, muscle and mechanical properties need not vary along the body for wave speed differences to emerge. It also shows that, during steady swimming, proprioceptive feedback is not necessary to produce this basic phenomenon. This supports the suggestion of Brown and Loeb that, in stereotypical movements, neural feedback (reflexes) can be partially or wholly replaced by mechanical feedback (called “preflexes” by Brown and Loeb (section 3 of [43]), who define a preflex as “the zero-delay, intrinsic response of a neuromusculo-skeletal system to a perturbation.”), and therefore might not be required for stability [43]–[45]. Further model-based and experimental support for this hypothesis has recently emerged in legged locomotion studies [15]. However, mechanosensitive “edge cells” exist within the lamprey's spinal cord, which can influence the timing of muscle force generation and phase relationships via feedback to the CPG and motoneurons [46]. This mechanism may account for the deficit in phase lags produced by the model (Figure 15), and it is are presumably important during changing conditions and maneuvers.

The muscle model we described in Methods cannot perfectly fit both the isometric and the sinusiodal forcing data. We chose to fit it to sinusoidal data with an activation-to-curvature phase difference of 0.1, close to values seen in the data and the control simulations. This is not ideal, and may influence the results described in the results section. We plan to extend the model to include secondary muscle properties responsible for the discrepancies in its predictions. Moreover, we have used a linear model for flexural stiffness (*M* = *EI*(*ϕ_s_*−*κ*), Equation 5), although the lamprey's body stiffness is nonlinear. More accurate estimates of body stiffness may also influence the results.

In our discretization the arms to which muscles are attached project perpendicularly from the center of each link toward the periphery (see Figure 4). In the lamprey, however, the myosepta to which the swimming muscles attach project obliquely backwards from the notochord toward the body wall so that the muscle layers interleave in a somewhat complicated fashion (albeit considerably less complicated than in bony fish; see [11]). We have not examined the consequences of this attachment geometry, but it can be expected to affect torques at the joints, and we intend to include it in a future study. It is of interest to note, however, that Katz et al. [47] have shown that in spite of more more complicated interleaving of muscle layers in teleost fish, the swimming muscles undergo length changes similar to those expected for a homogeneous, continuous beam, and that curvature of the midline gives a reliable measure of muscle length at any point along the body.

A further shortcoming of the present study, also noted in the methods section, is our use of an oversimplified model for fluid reaction forces. While Taylor's approximation in Equation 7 suffices for straight rods in uniform steady flow, it does not capture unsteady effects such as vortex shedding that are characteristic of swimming. These effects are likely important not only in creating propulsive thrust [22],[23], but the resulting reaction forces on the animal may also influence the speed at which the mechanical wave of curvature travels along its body. This would in turn affect the mechanical waves shown in Figure 11, perhaps changing the relative speed of activation and response. A more realistic model of vortex generation will also be needed to determine if negative work and tail stiffening are important in thrust generation, and to enable more definitive studies of swimming efficiency.

We also propose to use the present model, with the further addition of distributed CPG and motoneuron models [33],[48], to study proprioceptive feedback mechanisms in lamprey. In particular, it will allow us to investigate the influence of the aforementioned edge cells on the timing of muscle force generation. In recent experiments the isolated notochord/spinal cord preparation is rhythmically bent from side to side and the resulting edge cell feedback to motoneurons and CPG interneurons studied [49] (cf. [46]). This work complements our model in that it removes muscle activation, body elasticity and hydrodynamic forces, to reveal how an isolated sensory pathway can influence CPG phase and frequency relationships.
